# Metazoan parasite infection in the swordfish, *Xiphias gladius*, from the Mediterranean Sea and comparison with Atlantic populations: implications for its stock characterization

**DOI:** 10.1051/parasite/2014036

**Published:** 2014-07-25

**Authors:** Simonetta Mattiucci, Alexandra Garcia, Paolo Cipriani, Miguel Neves Santos, Giuseppe Nascetti, Roberta Cimmaruta

**Affiliations:** 1 Department of Public Health and Infectious Diseases, Section of Parasitology, “Sapienza” University of Rome P.le Aldo Moro, 5 00185 Rome Italy; 2 IPMA, I.P Avenida 5 de Outubro s/n 8700-305 Olhão Portugal; 3 Department of Ecological and Biological Sciences, Tuscia University Viale dell’Università s/n 01100 Viterbo Italy

**Keywords:** *Xiphias gladius*, Mediterranean Sea, Metazoan parasites, Fish stock

## Abstract

Thirteen parasite taxa were identified in the Mediterranean swordfish by morphological and genetic/molecular methods. The comparison of the identified parasite taxa and parasitic infection values observed in the Mediterranean swordfish showed statistically significant differences with respect to those reported for its Atlantic populations. A stepwise Linear Discriminant Analysis of the individual fish examined showed a separation among three groups: one including fish from the Mediterranean Sea (CTS, STS, and IOS); one consisting of fish from the Central South (CS), Eastern Tropical (ET), and Equatorial (TEQ) Atlantic; and a third comprising the fish sampled from the North-West Atlantic (NW); the CN Atlantic sample was more similar to the first group rather than to the other Atlantic ones. The nematodes *Hysterothylacium petteri* and *Anisakis pegreffii* were the species that contributed most to the characterization of the Mediterranean swordfish samples with respect to these Atlantic ones. *Anisakis brevispiculata*, *A. physeteris*, *A. paggiae*, *Anisakis* sp. 2, *Hysterothylacium incurvum*, *Hepatoxylon trichiuri*, *Sphyriocephalus viridis*, and their high infection levels were associated with the swordfish from the Central and the Southern Atlantic areas. Finally, *H. corrugatum*, *A. simplex* (s.s.), *Rhadinorhynchus pristis*, and *Bolbosoma vasculosum* were related to the fish from the North-West (NW) Atlantic area. These results indicate that some parasites, particularly *Anisakis* spp. larvae identified by genetic markers, could be used as “biological tags” and support the existence of a Mediterranean swordfish stock.

## Abstract (fr)

Treize taxa de parasites ont été identifiés dans les espadons de Méditerranée par des méthodes morphologiques et génétiques/moléculaires. La comparaison des taxons de parasites identifiés et des valeurs d’infections parasitaires observées dans les espadons de Méditerranée a montré des différences statistiquement significatives par rapport à ce qui a été rapporté pour les populations de l’Atlantique. Une analyse discriminante linéaire de chaque poisson examiné a montré une séparation en trois groupes : l’un qui comprend les poissons de la mer Méditerranée (CTS, STS et IOS), l’un composé de poissons des zones de l’Atlantique, zones Central Sud (CS), Tropical Est (ET) et équatoriale (TEQ), un troisième comprenant des poissons échantillonnés de l’Atlantique nord-ouest. L’échantillon Atlantique CN était plus similaire au premier groupe qu’aux autres groupes de l’Atlantique. Les nématodes *Hysterothylacium petteri* et *Anisakis pegreffii* sont les espèces qui ont le plus contribué à la caractérisation des échantillons d’espadons de la Méditerranée par rapport à ceux de l’Atlantique. *Anisakis brevispiculata*, *A. physeteris*, *A. paggiae*, *Anisakis* sp. 2, *Hysterothylacium incurvum*, *Hepatoxylon trichiuri*, *Sphyriocephalus viridis*, et leurs niveaux élevés d’infection étaient associés aux espadons des zones Centrale et Sud de l’Atlantique. Enfin, *H. corrugatum*, *A. simplex* (s.s.), *Rhadinorhynchus pristis* et *Bolbosoma vasculosum* étaient liés avec les poissons la région Nord-Ouest de l’Atlantique. Ces résultats indiquent que certains parasites, en particulier les larves d’*Anisakis* spp. identifiées par des marqueurs génétiques, pourraient être utilisés comme « marqueurs biologiques » et soutenir l’existence d’un stock d’espadon de Méditerranée.

## Introduction

The swordfish *Xiphias gladius* (Linnaeus, 1758) is a large pelagic fish distributed in tropical and temperate waters all over the world, including the Mediterranean Sea. In spite of its great migratory ability, the swordfish shows a homing behavior that maintains separate populations between and within the oceans [5–7, 15–17, 23, 29, 32, 38], as seems to be the case of the Mediterranean population where a separate stock has been considered [18, 34]. Genetic data based on both mitochondrial and nuclear markers agrees in showing that the Mediterranean population is highly differentiated from the North Atlantic one, with little or no gene flow [18, 34]. Moreover, it has a genetic structure characterized by a low genetic variability [18] and still bearing the signatures of both its ancient history and the last glacial maximum that occurred about 21–18,000 years ago [7]. Despite these reported homing behaviors, movements of swordfish have been reported, both for Atlantic specimens entering the Mediterranean Sea and for Mediterranean individuals feeding in the Atlantic waters close to the Gibraltar Straits [23].

In the Mediterranean region, including the Italian seas, the swordfish represents an important resource both as frozen and fresh seafood. Annual catch levels have fluctuated between 12,000 and 16,000 t in the last 15 years without any specific trend. These levels are relatively high and similar to those of larger areas such as the North Atlantic [3]. However, during the last 20 years the spawning stock biomass has declined markedly (between 24% and 38%), while the percentage of juveniles recovered in the catches has increased, suggesting that overfishing is occurring in the Mediterranean swordfish population [3, 72].

In the last few decades, studies aimed at fish population characterization in European waters have been fundamental in delineating programs for the sustainable exploitation of marine resources in the framework of a holistic approach [8, 60]. Many complementary methods were successfully applied, such as genetics, morphometrics, life-history traits and parasite surveys, coupled with appropriate statistics and modeling [1, 2, 19–21, 64]. In particular, parasites have been widely used in biological and ecological surveys of marine ecosystems [9, 48, 60]. Parasite surveys have provided important information about trophic web stability, water quality, global changes and host-population dynamics [43] of marine ecosystems, and the use of parasites as biological tags has become a useful tool in producing data for fish stock identification [10, 33, 36, 39–41, 44–47, 57–59, 67, 69, 70].

The parasite fauna of *X. gladius* and parasitic infection levels have been previously described for the Atlantic populations [13, 26, 30, 35] and the information gathered from parasite data supported the stock subdivision between the North and South Atlantic Ocean [27]. However, studies on the parasite fauna composition of the Mediterranean swordfish and its parasitic infection levels in comparison with the Atlantic populations are still lacking. The data acquired for this region so far include the description of new parasite taxa and the occurrence of parasitic infection by some ectoparasite species [25, 26, 54, 55].

In this study the infection levels by ecto-, meso-, and endoparasites in *X. gladius* from Italian waters of the Mediterranean Sea are reported. Moreover, the parasite fauna of the Mediterranean swordfish population was compared with that obtained from samples from the Atlantic Ocean gathered in our previous surveys [27, 28], to investigate differences which support the existence of an isolated swordfish population in the Mediterranean Sea.

## Materials and methods

### Fish sampling and methodologies

**Figure 1.**
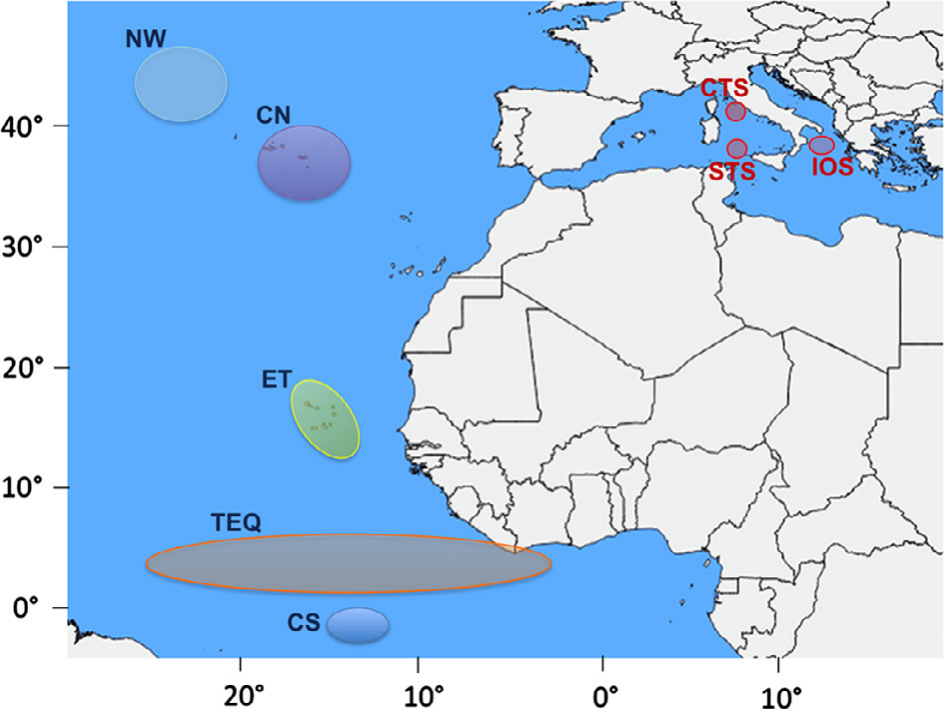
Sampling localities of the swordfish from the Mediterranean Sea examined in the present study in comparison with those of the Atlantic Ocean [27, 28]: Central Tyrrhenian Sea (CTS); South Tyrrhenian Sea (STS); Ionian Sea (IOS); and those from our previous studies [27, 28] from the Atlantic Ocean: North-West Atlantic (NW), Central North (CN), Eastern Tropical (ET), Tropical Equatorial (TEQ), and Central South (CS) Atlantic waters.

The parasitological survey was performed on 162 specimens of *X. gladius* from three different basin waters of the Mediterranean Sea, as reported in Table 1 and Figure 1. The fish were captured by pelagic long-line and trolling gear, between 2002 and 2004, from June to September. The lower-jaw fork length (LJFL) of the sampled specimens varied from 50 to 165 cm (Table 1), that corresponds, approximately, to 0- up to 4-year-old fish [9, 71]. Immediately after the capture, the LJFL was recorded from each fish on board the fishing vessels, and immediately, the swordfish body surface was visually inspected for the copepod *Pennella* spp.; the parasites found were removed from the fish muscle and stored in alcohol. The fish were then dissected on board, and the body visceral cavity was examined for the detection of the larval stage of anisakids and cestodes. These parasites were immediately stored in a frozen state or in alcohol. The stomach, intestine, and gills from each fish individual were removed, separated into plastic bags, numbered and stored in a frozen state at −20 °C, for further analysis. When on land, all the collected material was delivered, in a frozen state, to the Section of Parasitology (Department of Public Health and Infectious Diseases, Sapienza University of Rome) and stored there at −30 °C for the parasitological examination. Thus, conventional parasitological analysis was carried out on the gills, stomach, and intestine. All the metazoans were collected, counted, and preserved in 70% alcohol for the morphological identification, except for the larval anisakid nematodes, which were stored at −50 °C and then scored using multilocus allozyme electrophoresis analysis (MAE) for their species identification. MAE was used for the identification to the species level of 405 *Anisakis* spp. larvae, since they lack morphological diagnostic characters. The procedures used for the MAE are those previously detailed [50]. The diagnostic allozyme loci so far listed between the species of *Anisakis* [46, 50, 52] were investigated: adenylate kinase (*Adk-2*, EC 2.7.4.3), leucine-alanine peptidase (*PepC-1*, *PepC-2*, EC 3.4.11), and leucine-leucine peptidase (*PepB*, EC 3.4.11). In total, 405 larvae from the Mediterranean samples were identified to the species level.

**Table 1.** Number (*N*) of the examined *X. gladius*, during the years 2002–2004, fished in three localities of the Mediterranean, reported by size class (lower-jaw-fork-length), fishing gear, and sampling code.

Sampling locality	Code	Coordinates	<80 cm	80–109 cm	110–145 cm	>145 cm	Total *N*	Fishing gear
Ionian Sea	IOS	39°00′ N 18°00′ E	22	12	2	4	40	Long-line
Southern Tyrrhenian Sea	STS	38°06′ N 11°26′ E	3	7	9	18	37	Long-line and troll
Central Tyrrhenian Sea	CTS	41°59′ N 10°00′ E	30	28	26	1	85	Long-line
Total N			55	47	37	23	162	

### Data Analysis

The levels of infection were calculated for each parasite species and each sampling area of the Mediterranean Sea by means of the parameters of prevalence (P), mean intensity and its range (Im), and mean abundance (A). The analyses were carried out according to Bush et al. [12] and Rózsa et al. [63], by using the software QP3.0 [62]. The association between host size and mean intensity (MI) values was tested for the Mediterranean samples by Spearman’s correlation analysis (*r*_s_) [66].

The parasite fauna and the parasitic infection levels of the Mediterranean samples were compared with those from the Atlantic Ocean, previously examined by us [27, 28]. The swordfish Atlantic samples were from the following areas: North-West Atlantic (code: NW), Central North (code: CN), Eastern Tropical (code: ET), Tropical Equatorial (code: TEQ), and Central South (code: CS) Atlantic waters.

Chi-square analysis (χ^2^-test) and Kruskal-Wallis ANOVA (*H*) were performed to test for the significance values of the differences between prevalence and abundance estimates, respectively, of the infections observed by each parasite taxon in swordfish from the different sampling areas from the Mediterranean Sea, in comparison with those from the Atlantic Ocean. Values of *P* < 0.05 were considered statistically significant.

Among the parasite taxa recovered from the Mediterranean and the Atlantic swordfish samples, some were selected as potential biological markers for stock discrimination purposes according to the criteria given [40, 41, 69]. These parameters were: (1) presence/absence of parasite species in fish from the different geographic regions; (2) the finding of statistically significant differences in the infection levels (*P* and *A*) by the same parasite species in swordfish from the different geographical areas considered here; and (3) the parasite species should have a life span in the host long enough to cover the time scale of the investigation in the fish host. To test for differences in the relative proportions of larval *Anisakis* in swordfish specimens from the Mediterranean and Atlantic, a Principal Component Analysis (PCA) was performed. Finally, a stepwise Linear Discriminant Analysis (LDA) was performed to check for the differences between the parasite taxa selected and each sampling locality, to describe the differences among well-defined groups and to determine, among the selected parasites, which were responsible for dissimilarities among the fish samples. LDA analysis was computed after log (*n* + 1)-transformation of the number of each parasite taxon recovered by individual fish and areas. The importance of each taxon was assessed using a backward stepwise method based on Mahalanobis distances [68]. The analyses were performed by means of the Brodgar 2.5.1 package [11].

## Results

### Parasitic infection levels in the Mediterranean swordfish samples

A total of 13 taxa of metazoan parasites were recorded from the Mediterranean areas. According to the morphological analysis, the following taxa were recognized: the monogeneans *Tristoma coccineum* (Cuvier, 1817) and *T. integrum* (Diesing, 1850), the copepod *Pennella instructa* Wilson, 1917, a digenean of the genus *Prosorhynchoides* Dollfus, 1929, larval stages of the cestode *Hepatoxylon trichiuri* Holten, 1802 and of unidentified Tetraphyllidea, adults of the cestode species *Fistulicula plicatus* (Rudolphi, 1819), the nematode *Oncophora melanocephala* (Rudolphi, 1819) Baudin-Laurencin, 1971, and adults of the raphidascarid nematodes *Hysterothylacium corrugatum* Deardoff and Overstreet, 1981, *H. incurvum* (Rudolphi, 1819) Deardorff & Overstreet, 1981 and *H. petteri* Sheenko, 1991 (Table 2). Out of the 405 *Anisakis* spp. larvae identified genetically, 223 larvae from the fish from the IOS, 25 from the STS and 70 from the CTS were recognized as *A. pegreffii* Campana-Rouget and Biocca, 1955, based on diagnostic allozymes, while 80 larvae from swordfish sampled in the CTS, and 7 from the STS were assigned to the species *A. physeteris* Baylis, 1923 (Table 3).

**Table 2.** Parasitic infection levels of *X. gladius* from the Mediterranean Sea (present study, codes as in Table 1) and from the Atlantic Ocean (previously analyzed by [27, 28]).

Parasite taxa	Mediterranean Sea						Atlantic Ocean									
	CTS *N* = 85 mean size = 93.5		STS *N* = 37 mean size = 127.1		IOS *N* = 40 mean size = 87.1		NW *N* = 42 mean size = 127.9		CN *N* = 46 mean size = 106.7		ET *N* = 45 mean size = 129.8		TEQ *N* = 145 mean size = 159.41		CS *N* = 24 mean size = 148.5	
	*P*	MI ± SD	*P*	MI ± SD	*P*	MI ± SD	*P*	MI ± SD	*P*	MI ± SD	*P*	MI ± SD	*P*	MI ± SD	*P*	MI ± SD
Anisakis brevispiculata*	–	–	–	–	–	–	17	3.0 ± 3.3	–	–	36	5.8 ± 8.1	44	13.2 ± 14.1	13	4.7 ± 6.4
Anisakis paggiae*	–	–	–	–	–	–	–	–	–	–	2	1.0	2	1.0	17	1.0
Anisakis pegreffii*	37	2.4 ± 3.1	14	5 ± 3.6	53	64.2 ± 7.6	–	–	–	–	–	–	–	–	–	–
Anisakis physeteris*	28	3.7 ± 4.1	5	3.5 ± 3.1	–	–	21	2.1 ± 1.3	9	1.3 ± 0.5	44	7.5 ± 7.9	63	12.1 ± 14.6	17	6.0 ± 10.0
*Anisakis simplex* (s. s.)*	–	–	–	–	–	–	50	3.4 ± 1.3	22	4.0 ± 2.6	–	–	–	–	–	–
*Anisakis* sp.2*	–	–	–	–	–	–	–	–	–	–	20	3.8 ± 3.2	9	2.4 ± 1.4	–	–
Bolbosoma vasculosum*	–	–	–	–	–	–	31	1.5 ± 0.9	4	1.0	–	–	13	3.1 ± 2.6	–	–
*Fistulicola plicatus*	69	12.2 ± 5.8	82	7.7 ± 4.4	89	3.9 ± 3.1	91	5.1 ± 5.6	67	3.4 ± 2.8	82	7.7 ± 5.4	92	12.9 ± 10.2	100	16.1 ± 10.1
Hepatoxylon trichiuri*	1	1 ± 1	–	–	–	–	71	5.4 ± 6.8	57	5.6 ± 14.3	76	5.9 ± 6.7	75.2	4.1 ± 5.6	63	2.7 ± 1.8
*Hirudinella ventricosa*	–	–	–	–	–	–	41	1.8 ± 1.0	2	3.0	11	1.6 ± 0.9	21	1.5 ± 1.3	33	1.3 ± 0.5
Hysterothylacium corrugatum*	9	7 ± 27.5	24	6.3 ± 8.2	10	3.03 ± 32.5	100	25.7 ± 33.2	11	5.6 ± 5.4	91	18 ± 21.8	100	7.7 ± 10.6	71	14.1 ± 18.0
Hysterothylacium incurvum*	9	2.25 ± 57.3	24	11 ± 17.4	10	1.75 ± 89.2	100	18.6 ± 23.0	30	4.7 ± 5.3	91	54.5 ± 65.3	100	25.7 ± 35.4	79	39.6 ± 53.9
Hysterothylacium petteri*	8	6.7 ± 15.2	3	1 ± 34.9	3	1.04 ± 20.1	–	–	–	–	–	–	–	–	–	–
*Oncophora melanocephala*	2	1.5 ± 0.7	3	1.6 ± 0.4	–	–	2	1.0	4	1.0	–	–	1	1.0	–	–
*Pennella instructa*	100	5.5 ± 4.6	100	12.7 ± 18.7	100	5.2 ± 2.3	–	–	7	1.0	2	2.0	3	1.3 ± 0.5	–	–
*Prosorhynchoides* sp.	–	–	3	13 ± 5.5	3	40 ± 7.7	–	–	–	–	–	–	–	–	–	–
Rhadinorhynchus pristis*	–	–	–	–	–	–	43	4.1 ± 3.7	–	–	11	1.0	–	–	4	1
Sphyriocephalus viridis*	–	–	–	–	–	–	–	–	–	–	16	1.4 ± 0.5	11	1.1 ± 0.3	4	1.0
Tetraphyllidea spp.	8	1.9 ± 1.1	62	8.6 ± 4.6	11	12.5 ± 5.2	–	–	35	6.2 ± 9.5	–	–	5	2.4 ± 1.5	–	–
*Tristoma coccineum*	29	3.4 ± 2.7	30	5.9 ± 3.9	69	2.3 ± 2.4	60	7.5 ± 7.2	59	1.3 ± 0.6	18	2.1 ± 1.4	19	2.7 ± 2.3	13	1
*Tristoma integrum*	51	3.3 ± 3.0	47	4.1 ± 3.3	85	6.0 ± 3.7	76	7.9 ± 8.2	22	1.4 ± 0.5	80	6.4 ± 5.0	59	5.1 ± 5.1	54	3.5 ± 3.4

*P* = prevalence (expressed in percentage, %); MI = mean intensity; SD = standard deviation; *N* = number of swordfish examined. Parasites are listed in alphabetical order.

*Parasite taxa selected as potential biological tags for swordfish stock identification in the Mediterranean Sea and Atlantic Ocean; “–” no parasites recovered.

**Table 3.** Relative proportions of larval *Anisakis* spp. identified by genetic markers in the swordfish from the Mediterranean Sea (present study), compared with those sampled from the Atlantic Ocean (data from [25, 26]). *N* = number of *Anisakis* spp. larvae identified.

Sampling area	N	*A. pegreffii*	*A. simplex* (s.s.)	*A. physeteris*	*A. brevispiculata*	*A. paggiae*	*Anisakis* sp. 2
Mediterranean							
Sea IOS	223	100.0	–	–	–	–	–
STS	32	78.1	–	21.9	–	–	–
CTS	150	47.0	–	53.0	–	–	–
Atlantic Ocean							
NW	112	–	63.2	17.4	19.4	–	–
CN	45	–	89.0	11.0	–	–	–
CS	43	–	–	57.2	33.3	9.2	–
ET	277	–	–	54.0	33.4	0.4	12.2
TEQ	2367	–	–	43.0	52.2	0.4	5.0

No statistically significant differences were found in prevalence and mean intensity infection values in swordfish caught from the same Mediterranean area over the three years of study. Thus, the data were grouped and are presented by fish sampling area, in Table 2.

As for the pairwise comparison of the abundance values among the sampling areas of the Mediterranean Sea, statistically significant (*P* < 0.05) differences were observed for the larval nematodes *Anisakis pegreffii* and *A. physeteris*, and for the adult nematode *H. corrugatum* (Table 4). No significant differences (*P* > 0.05) in the abundance values were found in infection by the remaining parasite species between swordfish from different fishing grounds (Table 4). As for the copepod *Pennella instructa* – known as a mesoparasite [31] – all the Mediterranean swordfish samples were found to be parasitized by this mesoparasite at high prevalence (*P* = 100%) and MI values (up to 80 specimens collected in a single individual fish sampled in the STS area; Table 2). High prevalence values were observed in the case of infection by the adult cestode *Fistulicula plicatus* in all the Mediterranean swordfish samples (Table 2).

**Table 4.** Kruskal-Wallis ANOVA (*H*) and chi-square (χ^2^) pairwise tests of mean abundance (*A*) and prevalence (*P*) values of the infection by the selected parasite taxa of *X. gladius* from the Mediterranean Sea (areas: CTS, STS, IOS) versus the Atlantic Ocean (areas: NW, CN, CS, ET, TEQ). CTS, STS, IOS: data from the present study, TEQ: data from [27] NW, CN, CS, ET: data from [28]. Ab, Absent in both areas; **P < 0.01; *P < 0.05; NS = not significant, *P* > 0.05. Parasites are listed in alphabetical order.

Parasite taxa	Test	Overall	CTS vs. STS	CTS vs. IOS	CTS vs. NW	CTS vs. CN	CTS vs. CS	CTS vs. ET	CTS vs. TEQ	STS vs. IOS	STS vs. NW	STS vs. CN	STS vs. CS	STS vs. ET	STS vs. TEQ	IOS vs. NW	IOS vs. CN	IOS vs. CS	IOS vs. ET	IOS vs. TEQ
*Anisakis* sp.2	*H*	**	Ab	Ab	Ab	Ab	Ab	**	**	Ab	Ab	Ab	Ab	*	*	Ab	Ab	Ab	*	*
	χ^2^	**	Ab	Ab	Ab	Ab	Ab	**	**	Ab	Ab	Ab	Ab	*	*	Ab	Ab	Ab	*	*
*A. brevispiculata*	*H*	**	Ab	Ab	**	Ab	*	**	**	Ab	*	Ab	*	**	**	*	Ab	*	**	**
	χ^2^	**	Ab	Ab	**	Ab	**	**	**	Ab	*	Ab	*	**	**	*	Ab	*	**	**
*A. paggiae*	*H*	**	Ab	Ab	Ab	Ab	**	NS	NS	Ab	Ab	Ab	*	NS	NS	Ab	Ab	*	NS	NS
	χ^2^	**	Ab	Ab	Ab	Ab	**	NS	NS	Ab	Ab	Ab	*	NS	NS	Ab	Ab	*	NS	NS
*A. pegreffi*	*H*	**	*	*	**	**	**	**	**	**	NS	NS	NS	NS	**	**	**	**	**	**
	χ^2^	**	*	*	**	**	*	**	**	**	NS	NS	NS	NS	*	**	**	**	**	**
*A. physeteris*	*H*	**	NS	**	NS	**	NS	*	**	NS	NS	NS	NS	**	**	*	NS	**	**	**
	χ^2^	**	*	*	NS	*	NS	NS	**	NS	NS	NS	NS	**	**	*	NS	**	**	**
*A. simplex* (s.s.)	*H*	**	Ab	Ab	**	**	Ab	Ab	Ab	Ab	**	**	Ab	Ab	Ab	**	**	Ab	Ab	Ab
	χ^2^	**	Ab	Ab	**	**	Ab	Ab	Ab	Ab	**	*	Ab	Ab	Ab	**	**	Ab	Ab	Ab
*Bolbosoma vasculosum*	*H*	**	Ab	Ab	**	NS	Ab	Ab	**	Ab	**	NS	Ab	Ab	*	**	NS	Ab	Ab	*
	χ^2^	**	Ab	Ab	**	NS	Ab	Ab	**	Ab	**	NS	Ab	Ab	*	**	NS	Ab	Ab	**
*Hysterothylacium corrugatum*	*H*	**	*	*	**	NS	**	**	**	NS	**	NS	**	**	**	**	NS	**	**	**
	χ^2^	**	*	*	**	NS	**	**	**	NS	**	NS	*	**	**	**	NS	**	**	**
*H. incurvum*	*H*	**	*	NS	**	**	**	**	**	NS	**	NS	**	**	**	**	*	**	**	**
	χ^2^	**	NS	NS	**	*	**	**	**	NS	**	NS	*	**	**	**	NS	**	**	**
*H. petteri*	*H*	**	NS	NS	*	*	NS	*	*	NS	NS	NS	NS	NS	NS	NS	NS	NS	NS	NS
	χ^2^	**	NS	NS	*	*	NS	*	**	NS	NS	NS	NS	NS	NS	NS	NS	NS	NS	NS
*Hepatoxylon trichiuri*	*H*	**	NS	NS	**	**	**	**	**	Ab	**	**	**	**	**	**	**	**	**	**
	χ^2^	**	NS	NS	**	**	**	**	**	Ab	**	**	**	**	**	**	**	**	**	**
*Pennella instructa*	*H*	**	*	NS	**	**	**	**	**	NS	**	**	**	**	**	**	**	**	**	**
	χ^2^	**	NS	NS	**	**	**	**	**	NS	**	**	**	**	**	**	**	**	**	**
Rhadinorhynchus *pristis*	*H*	**	Ab	Ab	**	Ab	NS	**	NS	Ab	**	Ab	NS	*	NS	**	Ab	NS	*	*
	χ^2^	**	Ab	Ab	**	Ab	NS	**	NS	Ab	**	Ab	NS	NS	NS	**	Ab	NS	*	*
Sphyriocephalus *viridis*	*H*	**	Ab	Ab	Ab	Ab	NS	**	**	Ab	Ab	Ab	NS	*	*	Ab	Ab	NS	*	*
	χ^2^	**	Ab	Ab	Ab	Ab	NS	**	**	Ab	Ab	Ab	NS	*	NS	Ab	Ab	NS	*	*

Concerning the *Anisakis* spp. larvae genetically identified, the relative proportions of the larval nematodes recovered from the Mediterranean Sea differed across the three sampling areas. *A. physeteris* was only found in the samples from the Central (CTS) and Southern Tyrrhenian Sea (STS), while *A. pegreffii* was found at higher prevalence in the samples from the Ionian Sea (IOS) (Tables 2 and 3).

In terms of prevalence values by different size classes of Mediterranean swordfish (Table 2), statistically significant negative correlations (Spearman *r*_s_ = 0.03) were found in infection by the two monogenean species *Tristoma coccineum* and *T. integrum*; in particular, the fish of the size classes <80 cm were more infected by these parasites.

### Comparison of the Mediterranean Sea swordfish parasite fauna versus the Atlantic Ocean one: selection of parasites as biological tags

Univariate pairwise comparison of the parasitic infection levels by the parasite taxa identified from the Mediterranean swordfish samples with those observed in previous studies [27, 28] in the Atlantic ones (Table 4) revealed that most of the significant differences in the mean abundance (*A*) and prevalence (*P*) values occurred between the Mediterranean and Atlantic fish samples (Table 4). Indeed, high statistically significant differences in prevalence and mean abundance values were observed in infection by the larval cestode *Hepatoxylon trichiuri*, the copepod *Pennella instructa*, the larval stages of *Anisakis* spp. and the raphidascarid nematodes of the genus *Hysterothylacium* (Table 4). *Hepatoxylon trichiuri* showed a high percentage of infection in the Atlantic samples (*P*_NW_ = 71%; *P*_CN_ = 57%), while it appeared at very low prevalence in the Mediterranean ones (*P*_CTS_ = 1.2%). The opposite trend was observed for the copepod *Pennella instructa*, which had high prevalence (*P* = 100%) in all Mediterranean areas. On the contrary, in the fish from the Atlantic Ocean, the prevalence of infection by this crustacean was very low, with prevalence values of 7.0%, 2.0%, and 3% in the samples from the Central North (CN), Eastern Tropical (ET) and Tropical Equatorial (TEQ) areas of the Atlantic Ocean (Tables 2 and 4).

**Figure 2.**
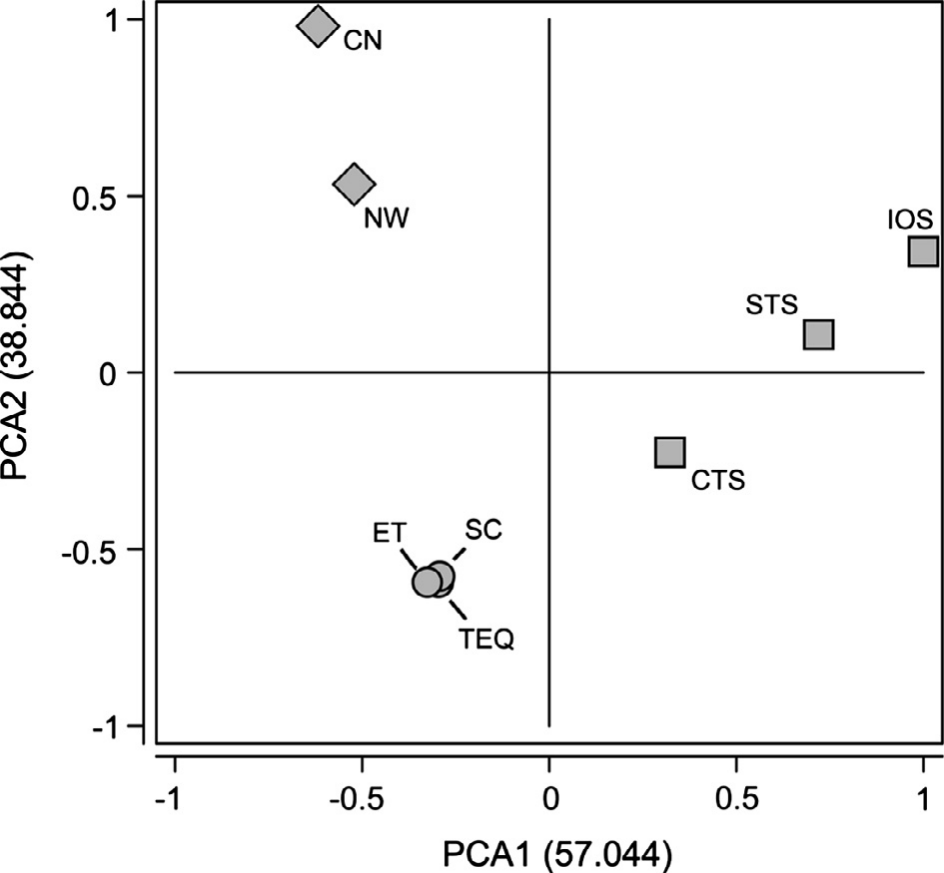
Principal Coordinate Analysis (PCA) of the swordfish specimens (*Xiphias gladius*) based on the distribution and infection levels of *Anisakis* spp. larvae identified by genetic markers shows how the first two axes account for about 57% and 39% of total ordination, respectively. Squares identify the Mediterranean samples, whilst circles and diamonds identify the Atlantic ones, where these latter identify the northernmost areas of the Atlantic Ocean waters. Codes for the sampling areas are the same as in Figure 1.

When comparing the *Anisakis* spp. larval distribution in the Mediterranean fish with respect to the Atlantic Ocean samples, *A. pegreffii* was the dominant species in the Mediterranean Sea fish, while *A. simplex* (s.s.) was the dominant species in the North Atlantic samples; it occurred in coinfection with *A. physeteris* in North-Western (NW) fishing grounds; and with *A. physeteris* and *A. brevispiculata* Dollfus, 1906 in fish captured from the Central North (CN) area (Table 3). However, *A. simplex* (s.s.) was absent moving to the *X. gladius* fished in the Central South (CS), Eastern Tropical (ET) and Equatorial (TEQ) Atlantic fishing grounds (Table 3). Indeed, the fish from the last three sampling areas were found to be parasitized by the most abundant larvae genetically recognized as *A. physeteris* and *A. brevispiculata* (Table 3). These two *Anisakis* species were found in coinfection in these fish with two other *Anisakis* species: *A. paggiae* Mattiucci, Nascetti, Dailey, Webb, Barros, Cianchi, Bullini, 2005, and a taxon indicated as *Anisakis* sp. 2 (Table 3), genetically distinct from the other *Anisakis* spp. detected so far [27, 51, 52]. Moreover, the different species of *Anisakis* also showed significant differences in their infection levels in the Mediterranean swordfish versus the Atlantic Ocean samples (Table 4). The PCA analysis based on the distribution pattern and infection levels with *Anisakis* spp. from swordfish showed the Mediterranean swordfish samples clustering separately from the Atlantic ones (Fig. 2). In addition, according to the larval distribution of genetically identified species of *Anisakis*, in the Atlantic waters, a cluster formed by the “northern” samples, well distinct from the cluster including the “southern” samples, was observed (Fig. 2).

**Figure 3.**
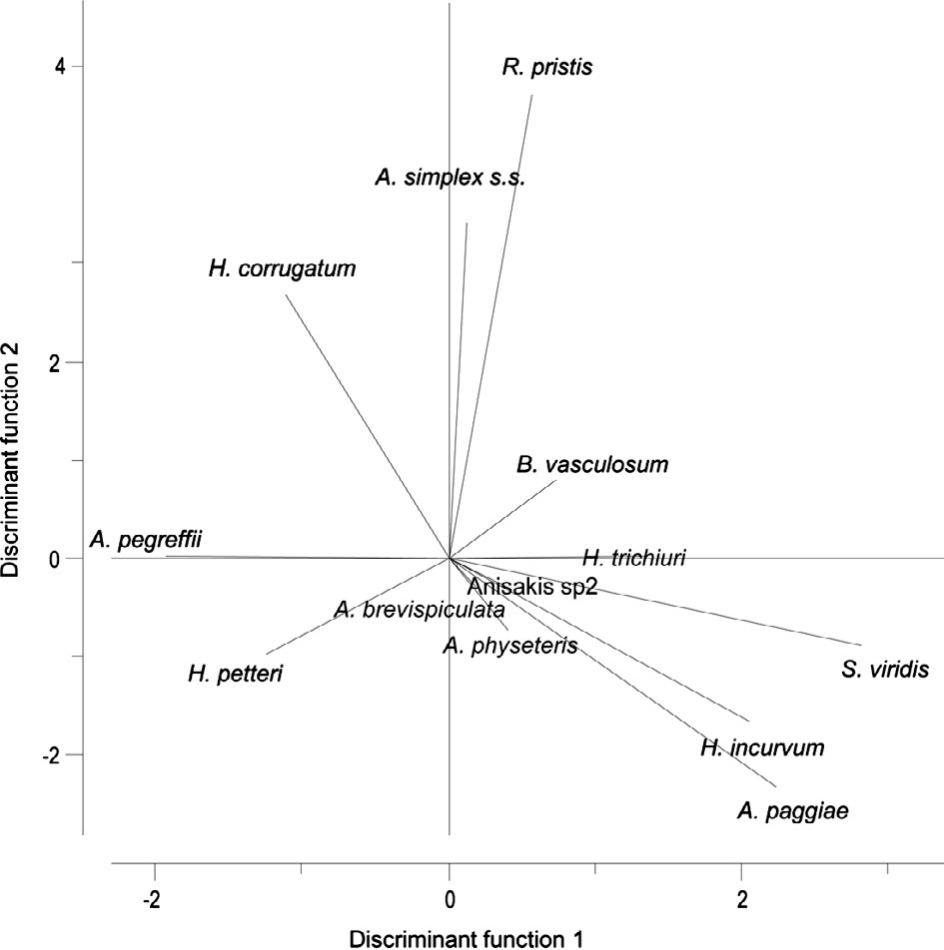
Linear Discriminant Analysis (LDA) based on the 13 selected parasites as biological markers of the Mediterranean samples (present study) versus the Atlantic ones (data from [27, 28]). Symbols represent mean values per area; each number represents single examined fish from each area: Star and (1): North-West (NW); Rectangle and (2): Central North (CN); Oval and (3): Central South (CS); Diamond and (4): Eastern Tropical (ET); hexagon and (5): Tropical Equatorial (TEQ); Square and (6): Central Tyrrhenian Sea (CTS); Triangle and (7): South Tyrrhenian Sea (STS); Circle and (8): Ionian Sea (IOS).

Thirteen parasites chosen, among the parasites detected, as potential biological tags helped in discriminating the Mediterranean population of *X. gladius* versus its Atlantic ones. The selected parasites included: two trypanorhynch species, *H. trichiuri* and *S. viridis*, two acanthocephalans, *R. pristis* and *B. vasculosum*, and the larval nematodes *A. pegreffii*, *A. simplex* (s.s.), *A. physeteris*, *A. brevispiculata*, *A. paggiae*, *Anisakis* sp. 2, and adults of *H. incurvum*, *H. corrugatum and H. petteri* (Table 2). The remaining taxa recovered from the Mediterranean samples were not considered as possible biological markers versus the Atlantic ones, due to the very low level of infection (*O. melanocephala*), unidentified species problems (*Prosorhynchoides* sp. and Tetraphyllidea spp.), their temporary presence in the fish host (*T. coccineum*, *T. integrum* and *F. plicatus*), and its high pathogenic role (*P. instructa*). In fact, the monogeneans *T. coccineum* and *T. integrum*, and the adult cestode *F. plicatus* have a short lifespan in swordfish, surviving less than 1 year [67]. The multivariate LDA (Fig. 3) carried out on those parasites (*n* = 13) chosen as biological markers of Mediterranean and Atlantic swordfish populations (Table 2) showed that the first two discriminant functions explained 81.2% of the variance, accounting for 47.3% (eigenvalue = 1.793) and 33.9% (eigenvalue = 1.287), respectively. A statistically significant group effect was found (Wilks *λ* = 0.081, *F*_15.106_, *P* < 0.01). The spatial distribution of individual fish, represented by the sample scores and the group means, showed a separation among three distinct groups (Fig. 2): (1) fish from the Mediterranean Sea (CTS, STS, and IOS); (2) fish from the North-West (NW) Atlantic, with fish from the Central North area (CN) being more similar to the Mediterranean Sea samples (CTS, STS, and IOS) rather than to those from Atlantic ones; and, finally, (3) fish from the Southern Atlantic areas (ET, TEQ, CS) (Fig. 3).

**Figure 4.**
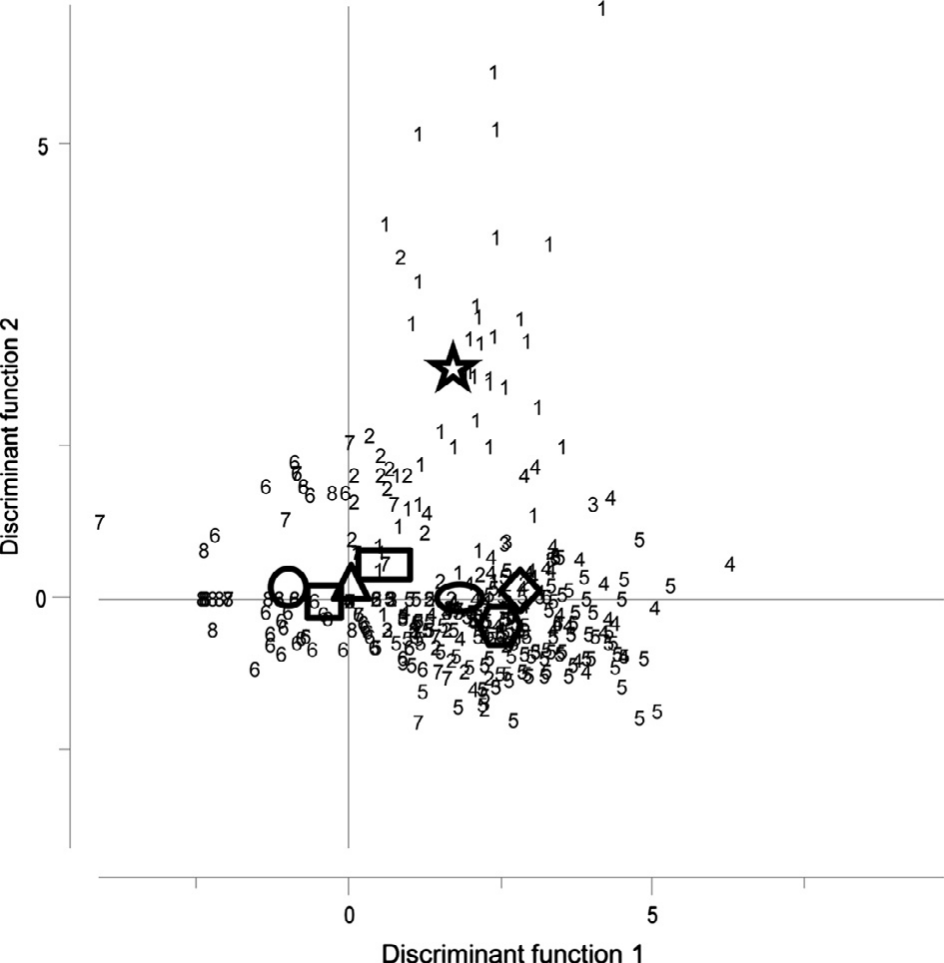
Discriminant analysis comparing the thirteen parasites selected as potential biological markers in Mediterranean versus Atlantic swordfish samples.

Among the selected parasites as biological markers, *Hysterothylacium petteri*, and *Anisakis pegreffii* were the species that mostly contributed to the distribution of samples from the Mediterranean Sea, being related to the fish captured in the CTS, STS, and IOS areas (Fig. 3). Additionally, *Hysterothylacium incurvum*, *A. brevispiculata*, *A. paggiae*, *Anisakis* sp. 2, A. *physeteris*, *Hepatoxylon trichiuri*, and *Sphyriocephalus viridis* (Wagener, 1854) were associated with the swordfish from the southern Atlantic areas (ET, TEQ, CS) and Central North (CN). Moreover, *H. corrugatum* was inversely correlated with *A. paggiae* and *H. incurvum* and related to the fish from the NW Atlantic area, as well as *A. simplex* (s.s.), and the acanthocephalans *Rhadinorhynchus pristis* (Rudolphi, 1819) and *Bolbosoma vasculosum* (Rudolphi, 1819) (Table 4 and Fig. 3).

According to the results achieved by the multivariate analysis, a second LDA was computed considering three groups: (i) the three sampling areas from the Mediterranean Sea (CTS, STS, and IOS) and the CN from the eastern Atlantic waters; (ii) the ET and TEQ from the eastern Atlantic waters and the CS area from the South Atlantic waters; and (iii) the NW Atlantic area. Re-analysis of the data resulted in substantial improvement in discrimination as the two discriminant functions now explained 100% of the variance, the first accounting for 54.9% (eigenvalue = 1.466) and the second for 45.1% (eigenvalue = 1.203). A statistically significant group effect was also found (Wilks *λ* = 0.184, *F*_45.972_, *P* < 0.01). Classification into three categories also had a strong effect on the accuracy of correctly classified samples that was improved to 84.3%.

## Discussion

Swordfish parasite fauna and infection levels were previously described from the Atlantic area [13, 27, 28, 30]; some data on its parasites from the Mediterranean Sea waters were also described previously [25, 51, 54, 55]. This study documents additional data on the infection levels of all ecto-, meso-, and endoparasites of the swordfish from the Mediterranean Sea and the data obtained were used for the first time to compare the Mediterranean population of *X. gladius* with its Atlantic ones.

All the parasite species recorded were already known for the host species. However, the results obtained in the present study highlight differences in the occurrence of some parasites detected in the fish host from different fishing grounds of the Mediterranean Sea versus those from the Atlantic Ocean. These differences are mainly related to the biogeographical aspects of some of the parasites species detected, and their life cycle. In other cases, the host body size may explain some of the differences observed, such as in the reduction of the infection by *Tristoma* spp., characterized by a direct life cycle, observed in the larger fish (>50 kg); this finding was already documented and likely related to abiotic factors and/or host behavior [25, 35, 54, 55].

The copepod *Pennella instructa* showed very high levels of infection in the Mediterranean swordfish, quite similar to those previously found on the same fish species captured from the eastern Mediterranean Sea [54, 55, 57]. On the contrary, this mesoparasite was present at a very low rate in the samples from the Central North and Eastern Tropical and Equatorial waters of the Atlantic Ocean. It was absent in all the other examined Atlantic samples [27]. These parasites are considered to be “mesoparasitic”, since the thorax and abdomen become deeply embedded within the host’s tissues, whereas the genital segment protrudes externally and bears egg sacs. Once attached to the final hosts, females exhibit gigantism as a result of massive expansion and, in some species, the production of a substantial holdfast. A very deep penetration of the mesoparasite reaching well into the body cavity and invading the circulatory system, including the heart, has been observed in the Mediterranean swordfish examined in the present study. Granulomas surrounding the holdfast of the copepod were found in the flesh and in the visceral body cavity of fish infected by *P. instructa*; in some of the removed granulomas, the destroyed holdfasts of the parasite, as the result of a past infection, were also observed. The parasite could affect the fitness of the fish host, also leading to the reduction of the muscle mass [30]. As a consequence, this parasite could also have a negative impact on the commercial value of this fish species.

Although still controversial [73], it has been suggested that when a host population shows a low genetic variability – as a consequence of its demographic reduction – it may be much more prone to pathogens, including a high parasitic burden [4, 37, 61]. Given the low level of the genetic variability so far recorded in the Mediterranean population of swordfish [18], and the parasitic burden due to *P. instructa* observed in the present study, a future investigation could be devoted to looking for a possible relationship between swordfish genotypes/haplotypes and susceptibility/resistance to *Pennella instructa.*

Most of the selected parasite species used here as tags in the comparison between the Mediterranean and Atlantic samples are transmitted via trophic webs and can be accumulated in the host overtime. In fact, in order to consider a parasite as an effective population marker, its residence time in the fish host should be as long as its fish host survival. Lester et al. [36] distinguished between permanent, semi-permanent, or temporary parasites. In the present study, 13 taxa were considered permanent or semi-permanent, because they have a long lifespan in the swordfish [14, 33, 67] and, thus, they were used as biological tags in the comparison between the Mediterranean and Atlantic swordfish populations.

The species of the genus *Hysterothylacium* do not survive as long as the life span of the fish host; they are luminal parasites and, as a consequence, they should be considered as transient parasites. However, the occurrence of different species of *Hysterothylacium* and their patterns of infection provided useful clues to characterize Mediterranean versus Atlantic populations of *X. gladius*, the species *H. petteri* being recorded, in the present study, only from the Mediterranean Sea. Further, the high infection levels reported here for the species *H. incurvum* and *H. corrugatum* suggest that they are dominant and likely specialist parasites for this fish species, *X. gladius* being reported as the definitive host of these species of *Hysterothylacium* [24]. On the other hand, Mackenzie et al. [41] found the larval nematodes of *Anisakis* spp. and *Hysterothylacium aduncum* as the most effective tags to distinguish a Northern stock from the North Sea of the horse mackerel, *Trachurus trachurus*, in European waters [41]. Similarly, cod (*Gadus morhua*) populations from the South-Eastern and South-Western Gulf of St. Lawrence from the Western Atlantic Ocean were classified as two separate stocks just based on the results of a multivariate analysis carried out on the nematodes *Pseudoterranova decipiens* and *Hysterothylacium aduncum* [53].

While the swordfish is the definitive host in the life cycle of the species of *Hysterothylacium* identified in the present study, *X. gladius* should be considered as an accidental host in the life cycle of the larval nematodes *Anisakis* spp. Indeed, it just acquires the infection by *Anisakis* spp. larvae by preying on fish and squid likely infected by these larval parasites; *X. gladius* likely represents a “dead route” in the life cycle of *Anisakis* spp., not being a prey item of cetaceans.

Just concerning the *Anisakis* species, the relative proportions of the larvae identified in the present study, in comparison with previous reports [27, 28], support the idea that the Mediterranean population of *X. gladius* is separated from the Atlantic ones.

In fact, *A. pegreffii* was identified only in *X. gladius* from the Mediterranean Sea fishing grounds. On the contrary, larvae of *A. brevispiculata*, *A. paggiae*, *Anisakis* sp. 2, and *Anisakis simplex* (s.s.), were absent in *X. gladius* sampled from the same Mediterranean waters. This finding is in accordance with the geographic distribution reported for *A. pegreffii*, being the most prevalent species of the genus parasitic on fish and cetaceans of the Mediterranean Sea [48]. The higher proportion of *A. pegreffii* found in the present study in the swordfish sampled from the Ionian Sea with respect to the other areas of the Mediterranean Sea could be related to the fact that this water basin is inhabited by a high density of various dolphin species, which constitute the main definitive hosts of this species. *A. pegreffii* was also found in coinfection with *A. physeteris* genetically recognized in the samples from the Central (CTS) and Southern Tyrrhenian Sea (STS). *A. physeteris* was previously documented in the western Mediterranean Sea [45]. On the other hand, *A. physeteris* was found in high proportion and showed higher infection levels in *X. gladius* fished in Eastern Tropical and Tropical Equatorial Atlantic waters, with respect to those observed in the Mediterranean samples. Indeed, in these Atlantic fish, the species *A. physeteris*, *A. brevispiculata*, *A. paggiae*, and *Anisakis* sp. 2 are the dominant taxa in *X. gladius.* These findings are in accordance with the geographical distribution reported so far for these species. In fact, *A. physeteris*, *A. brevispiculata*, and *A. paggiae* have so far been found in cetaceans belonging to the family Physeteridae of the Atlantic and Pacific Ocean waters [22, 48, 49]; it has also been suggested that the life cycle of these *Anisakis* spp. likely involves squid, rather than fish [48], as they represent the favorite prey items of Physeteridae. The finding of a high percentage of these larval *Anisakis* spp. in swordfish from the Atlantic could be related to the fact that swordfish and sperm whales share the same food source in this oceanographic area.

Thus, based on the results achieved on the different species of the larval *Anisakis* recognized in *X. gladius* from the Mediterranean and Atlantic fishing grounds, analyzed here, different populations of swordfish were distinguished (Fig. 2). These findings confirm that *Anisakis* spp. larvae, genetically recognized, can be used as biological tags of the fish population origin, as previously shown in a multidisciplinary stock analysis of pelagic (for instance, the horse mackerel, *Trachurus trachurus*) and demersal (for instance, the European hake, *Merluccius merluccius*) fish species from the Mediterranean versus the Atlantic waters [1, 2, 19, 21, 45–47]. Thus, larval nematodes of *Anisakis* spp. emerged in this study as effective biomarkers to distinguish fish population units considering the spatial structure of *X. gladius*. Indeed, the pattern of distribution of *Anisakis* spp. found in *X. gladius* reflects the different geographical distribution of these *Anisakis* spp. and, thus, it satisfies the assumption that the data observed are related to differences in the fishing grounds where the fish have been sampled.

According to Mackenzie and Abaunza [40] and Mackenzie [39], another criterion to consider a parasite species as a potential marker is also its infection level, which could be different among the fish sampling areas. In this study, the multivariate analysis of the distinctive pattern of infection with the nematodes *A. pegreffii* and *H. petteri* allowed us to discriminate the Mediterranean swordfish unit from those of the Atlantic Ocean. Similarly, while *Hepatoxylon trichiuri* was found in a single individual from the Mediterranean area, its occurrence at a high rate in fish from the Atlantic areas supports the same evidence. The same finding does not seem to support the hypothesis that fishes from the Atlantic enter the Mediterranean Sea since the single individual of *X. gladius* parasitized by *H. trichiuri* was not infected by other parasites “typical” of the Atlantic samples, such as *A. simplex* (s.s.), *A. brevispiculata*, *A. paggiae*, and *Anisakis* sp. 2.

The swordfish represents an important seafood source worldwide and, therefore, an assessment of stocks’ boundaries is important to implement its sustainable exploitation, as signaled, for example, by the increasing proportion of juveniles in the catches of the Mediterranean stock [71]. Overall, the multivariate analysis and the pattern of infection observed in those parasite species chosen as markers supports the evidence that the swordfish Mediterranean population of *X. gladius* is clearly distinct from its Atlantic populations units. This finding is in agreement with the data on the swordfish genetic structure, reporting that the Mediterranean swordfish stock is genetically highly divergent from the others, including the North-Atlantic ones [15–17, 34, 42, 65]. This is likely due to the ancient history of this species, surviving in isolation in the eastern Mediterranean basin during the last two glacial events and still poorly exchanging genes with the Atlantic populations, due to its phylopatric behavior [6]. Interestingly, while a genetic substructuring of *X. gladius* from the Mediterranean Sea stock has been revealed by phylogeographic analysis of the mitochondrial control gene [74], supporting the existence of an “eastern” and a “western” swordfish population unit, the parasite markers do not seem to be in agreement with this finding. However, the significantly different infection pattern with *Anisakis pegreffii* and the absence of *A. physeteris* in the swordfish sampled from the Ionian Sea with respect to the Central and Southern Tyrrhenian Sea suggest a further investigation of swordfish from other parts of the eastern Mediterranean Sea, searching for a possible accordance with the evidence of a differential spawning and reproductive region in the Mediterranean Sea.

The genetic data sets produced so far on *X. gladius* recognize the existence of a “northern” and a “southern” Atlantic stock. The parasitological data agree with the genetic results gathered on the fish host when describing the existence of two discrete stocks of the fish species in the Atlantic Ocean. Indeed, the multivariate analysis on the parasites selected as biological tags showed that, in the Atlantic, the North-Western (NW) sample was characterized by the patterns of infection with *Hysterothylacium corrugatum* (s.l.) and *A. simplex* (s.s.), while *A. paggiae*, *A. brevispiculata*, *Anisakis* sp. 2, *H. incurvum*, and *S. viridis* were the most effective biological tags distinguishing the “southern” Atlantic stock of *X. gladius*. However, when defining the boundary between the two Atlantic stocks, the results obtained by different genetic/molecular markers on *X. gladius* are discordant with each other, either confirming the 5° N position [32], or moving it North to 5° N [17] or evidencing an intergradation zone in the equatorial area [29]. According to the parasite markers, while the distinction between the NW swordfish stock from the remaining Atlantic populations – clearly supported by the multivariate analysis – is in accordance with the genetic data, the boundary between the NW and the “southern” stock seems to be less certain. Indeed, the multivariate analysis revealed that the Central, Tropical, and Equatorial Atlantic populations of swordfish are quite similar to each other. This suggests a possible boundary between the northern and southern stocks, northern to 5° N, or at least, the data are in accordance with the existence of a mixing area in the Tropical Equatorial Atlantic waters [29].

While the North Atlantic swordfish recently benefited from the management actions that rebuilt its stock consistency [56], on the contrary, the Mediterranean swordfish is still in need of effective management to counteract the loss of its spawning stock biomass and the ever-increasing percentage of juveniles in its landings, signaling a possible stock collapse into the next generation time of 7–10 years [71, 72]. Therefore, the parallel monitoring of the parasitic infection levels of this fish species in the Mediterranean Sea will represent an important aspect in addressing the fish stock’s “health state” in the framework of a holistic approach in fisheries management [8].
